# Changing distribution patterns of canine vector borne diseases in Italy: leishmaniosis vs. dirofilariosis

**DOI:** 10.1186/1756-3305-2-S1-S2

**Published:** 2009-03-26

**Authors:** Domenico Otranto, Gioia Capelli, Claudio Genchi

**Affiliations:** 1 Department of Veterinary Public Health and Veterinary Sciences, Faculty of Veterinary Medicine, University of Bari, Valenzano (Bari), Italy; 2 Istituto Zooprofilattico Sperimentale delle Venezie, Legnaro (PD), Italy; 3 Department of Veterinary Pathology, Hygiene and Public Health, University of Milan, Milan, Italy

## Abstract

Ecological and climatic changes, human and animal population dynamics are among the several factors that have favoured the spread or the (re)introduction and establishment of "novel" vector species and pathogens they transmit in previously disease-free geographical areas. As key examples of the changing pattern of distribution of canine vector borne diseases (CVBDs), the current distribution of canine leishmaniosis (CanL) by *Leishmania infantum *and dirofilariosis by *Dirofilaria immitis *causing heart worm disease (HW) in Italy is discussed on the basis of retrospective historical reports until the 90's and later on until 2009. For long time, *D. immitis *has been considered mainly present along the Po River Valley and northward areas, while *L. infantum *in south-central Italy and Sicily and Sardinia. Comparison of current available and historical data (up to 1989) confirms that HW and CanL, although with different prevalence rates, have been changing their distribution patterns in Italy as a result of many biological and ecological factors, including those related to vector distribution and introduction of new species (e.g. the Asian tiger mosquito *Aedes albopictus*, a competent vector of *D. immitis*). New autochthonous foci of HW in southern Italy (*i.e*. Apulia and Calabria regions) have recently been reported.

Although analysing retrospective data may represent a difficult task, the "paradigm" about the dual distribution of HW and CanL in northern and southern Italy cannot yet be considered valid. The research needs for managing HW and CanL in previously uninfected areas are discussed.

## Background

The global spread of parasitic arthropods and of canine vector borne diseases (CVBDs) have no more boundaries across the planet. The combination of several factors (e.g. ecological and climatic changes, human and animal population dynamics) may affect, to different extents, the occurrence and spread of CVBDs in different geographical areas [1,2]. Movement of people (for tourism, work, etc.) and increased exchange of goods through a range of types of transportation (e.g. containers, aircraft cargoes) may play a crucial role for the (re)introduction and establishment of "novel" vector species and pathogens in previously disease free areas [3]. Such was the case of the Asian tiger mosquito *Aedes albopictus *which is a competent vector of *Dirofilaria immitis *to dogs [4] and of West Nile virus, Japanese B encephalitis, chikungunya virus and other arboviruses to humans [5]. This mosquito species has successfully spread through many areas of the world, including Italy, causing outbreaks of vector borne infections in animals and humans [5,6].

However, the spread into a previous non-endemic geographic area of vectors and of pathogens they transmit may be favoured and facilitated by arthropod non-specific host preferences, biological life cycle, off-host ecology, feeding behavior, presence of competent hosts and adaptability to different environmental conditions [7-9]. Many arthropods (e.g. ticks, sandflies and mosquitoes) infest dogs both in urban and rural areas, being adapted to survive in indoor and outdoor environments, increasing the risk of emergence or re-emergence of certain metazoonotic diseases [2]. Obviously the occurrence and establishment of a CVBD is regulated by a complex chain of interactions among pathogens, vectors and the environment.

Historically, the distribution of canine leishmaniosis (CanL) by *Leishmania infantum *and dirofilariosis by *D. immitis *in Italy was considered to be "dual", being *D. immitis *mainly present along the Po River Valley and northward areas [10] and *L. infantum *in south-central Italy and Sicily and Sardinia [11]. As a consequence, for long time, clinicians and parasitologists living in southern and northern Italy have been more used to deal with CanL and dirofilariosis respectively. As key examples of changing pattern of distribution of CVBDs, here we discuss the current distribution of these diseases, which are regarded among the most important and severe CVBDs of zoonotic concern. More specifically, we describe the occurrence of autochthonous foci of dirofilariosis by *D. immitis *in southern Italy and of recently detected foci of *L. infantum *in northern Italy, discussing scenarios of changing distribution patterns of both infections throughout this country.

## Distribution of dirofilariosis and leishmaniosis in Europe

**Figure 1:**
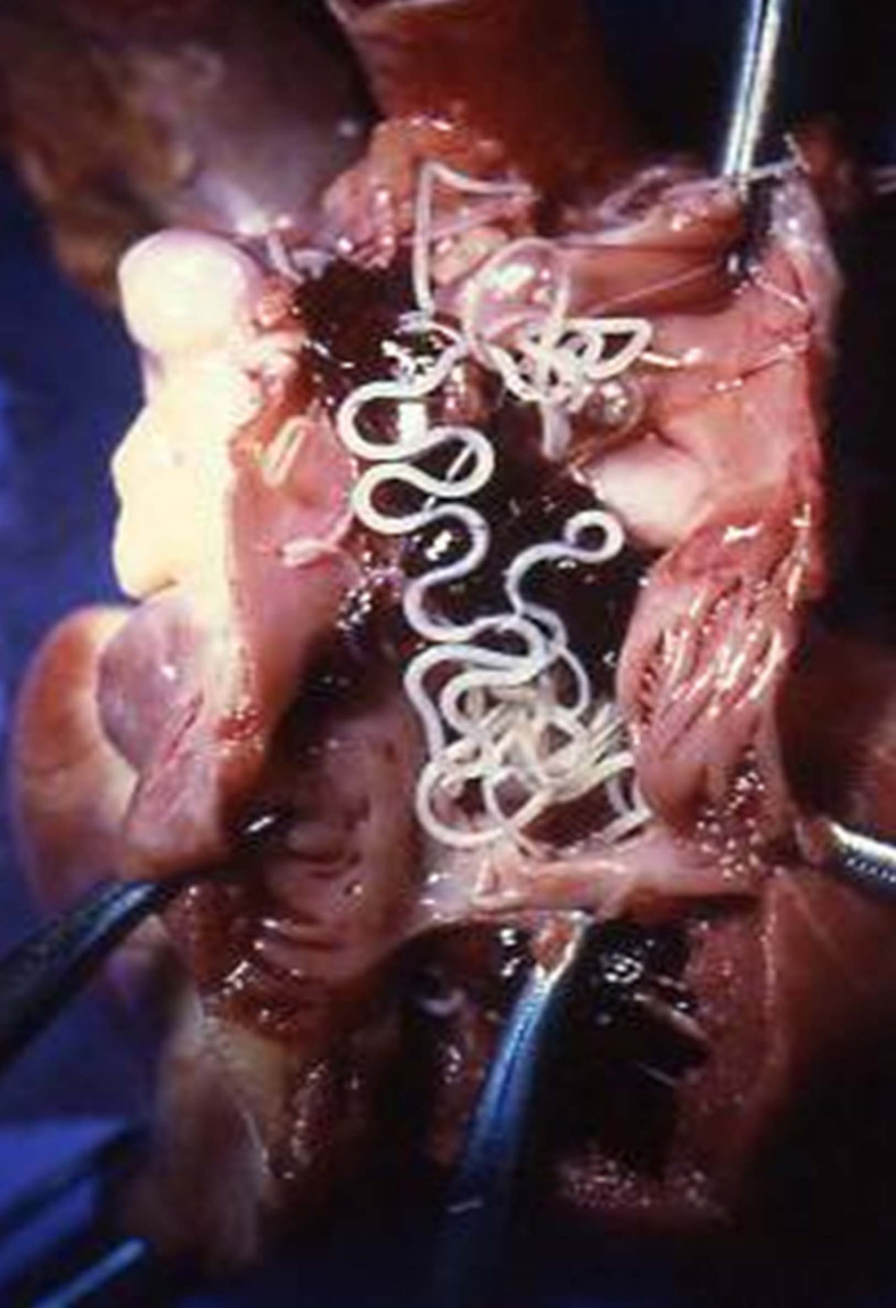
**Adult *Dirofilaria immitis *in the heart of a dog**. *Dirofilaria immitis *adult worms at the dissection of a heart of a dog.

*Dirofilaria immitis *and *Dirofilaria repens *represent the most important filarial species in Europe both because of their pathogenicity on dogs' health and because of their zoonotic potential [12]. *D. immitis *is the causative agent of heartworm disease (HW) (Figure 1) which is widespread through tropical and temperate regions of the world, while *D. repens *causes a less pathogenic form, infesting subcutaneous tissues. Cases of human pulmonary dirofilariosis have been increasingly reported worldwide [13]. Indeed, *D. repens *adult worms occur in subcutaneous tissues [less frequently *Acanthocheilonema *(syn. *Dipetalonema*) *reconditum, Acanthocheilonema *(syn. *Dipetalonema*) *grassii *and *Acanthocheilonema *(syn. *Dipetalonema*) *dracunculoides*] or in the heart (*D. immitis*) of mammals (mainly primates and carnivores) and they are transmitted, as infective third stage larvae, by mosquitoes (*Dirofilaria *spp.) and other arthropods such as flies, lice and ticks [13].

Although dirofilariosis has for long time been considered a disease of veterinary concern and HW in dogs and cats might be a severe and often fatal disease in untreated animals, *D. repens *has been until recently recognized as an emerging metazoonosis in Europe (e.g. Italy [14,15]. Indeed, *D. immitis *is endemic in southern regions of Europe even if, in the last decades, the number of reports in northeastern countries (e.g. Czech Republic, Serbia and Slovak Republic) is increasing [16-22]. Though the distribution of *D. repens *in Europe is less studied probably because of its reduced pathogenicity, this species is present in Italy, France and the eastern European countries [22].

**Figure 2:**
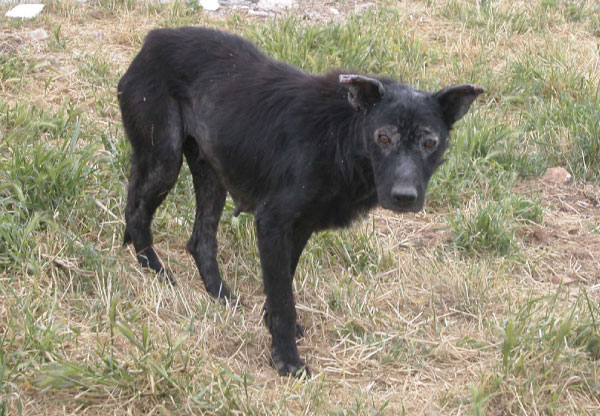
**Dog showing severe clinical signs of leishmaniosis**. A dog from southern Italy positive for *Leishmania infantum *both at the parasitological and serological tests presenting poor general conditions.

*Leishmania infantum*, the causative agent of CanL in Mediterranean areas (Figure 2), is transmitted by different species of phlebotomine sandflies within the genus *Phlebotomus *[23,24]. The disease is considered to be one of the most important CVBDs of zoonotic concern, being widely distributed in Europe. CanL is endemic along the Mediterranean coast, from Portugal to Turkey, including Cyprus and Crete [3]. In this area, the prevalence of *L. infantum *infection varies widely, but might be as high as 80% [25]. More recently, infection by *L. infantum *in dogs has spread through northern Italy [26] and some countries of central Europe [27]. While in non-endemic areas, *L. infantum *infection mainly causes severe clinical forms [26], in endemic areas, most of the affected dogs may remain asymptomatic [28]. Nonetheless, asymptomatic animals might play a role in maintaining the infection in an endemic area by transmitting the infection to dogs and other receptive hosts including humans [29].

## Dirofilariosis and leishmaniosis: two diseases for two "*Italys*"

For its geographical position (between 47°–36° north) and elongated shape, Italy may be represented as two countries. In particular, the Italian peninsula presents a large variety of habitats and environments, from the northern Alps through the Apennine Mountains to southern Italian and island regions. Accordingly, most of the inland northern areas have a continental humid climate whereas the coastal areas of the Liguria region and most of the peninsula, a Mediterranean temperate climate. The coastal regions have mild winters and warm and generally dry summers, although lowland valleys can be hot in summer.

The geographical distribution of dirofilariosis and leishmaniosis in northern and southern Italy respectively, although anecdotal, has reflected for a long time the different habitats and distribution of arthropod vectors. While the Culicidae vectors of *D. immitis *are mainly diffused and they develop during the summer months in humid climate areas of northern Italy [30], the coastal areas of the Liguria and the Mediterranean temperate regions of central and southern Italy are optimal to the development of different species of sandflies [31].

In Italy, canine dirofilarioses are transmitted by a number of culicid species (Table 1), which are mainly active during the late spring and summer [32]. The role of different Culicidae spp. as vectors of *Dirofilaria *spp. has been investigated by using insect dissection and molecular methods (Table 1) and their vectorial competence has been ascertained or suspected on the basis of the finding of DNA in different anatomical parts of mosquitoes [33].

**Table 1:** Proven or suspected Culicidae vectors of *Dirofilaria immitis *and *Dirofilaria repens *in Italy (North -N; South -S; Sicily -Si; Sardinia -Sa) [4,33,50].

	Distribution	*D. immitis*	*D. repens*
*Anophelinae*			
Anopheles claviger*	N, S, Si, Sa	-	-
*A. maculipennis*	N, S, Si	mol./f.c.s.	mol./f.c.s.
*Culicinae*			
*Aedes albopictus*	N, S, Sa	mol./f.c.s.	mol./f.c.s.
Ae. cantans*	N	-	-
Ae. caspius*			
*Ae. cinereus*	N, S, Si, Sa	-	-
	N	mol./abd.	-
*Ae. geniculatus*	N, S, Si, Sa	mol./abd.	-
*Ae. detritus*	N, S, Si, Sa	mol./abd.	-
*Ae. punctor*	N, S	mol./abd.	-
Ae. vexans*	N, S, Si, Sa	-	-
Coquillettidia richiardii*	N, S, Si, Sa	mol./f.c.s.	-
Culiseta annulata*	N, S, Si, Sa	-	-
*Culex modestus*	N, S, Si, Sa	mol./abd.	-
*C. pipiens*	N, S, Si, Sa	mol./f.c.s.	mol./f.c.s.
C. territans*	N, S	-	-
*C. torrentium*	N	mol./abd.	-

* Culicidae spp. reported in Italy and suspected vectors of *Dirofilaria *spp. [33]. Evidence about the competence of Culicidae as vectors of *Dirofilaria immitis *and/or *Dirofilaria repens *in Italy relies on laboratory or field capture studies by dissection or PCR testing of field collected samples (mol./f.c.s.). PCR positive results of abdomens (mol./abd.), which however do not prove vector competence are also reported [4,33,50].

The most common filarial species parasitizing dogs in Italy are *D. immitis *and *D. repens *(less frequently *A. reconditum, A. grassii *and *A. dracunculoides*). Canine dirofilariosis caused by *D. immitis *is considered endemic in northern regions with prevalence rates ranging from 22 to 80% [16,34]. However, while *D. repens *was mainly widespread in southern regions, with the exception of Piedmont region in the north [34], the classical hyperendemic areas for *D. immitis *were along the Po River Valley [17]. The distribution and prevalence of dirofilariosis has been also studied in dogs from central and northern Italy, while epidemiological data on the occurrence of dirofilariosis by *D. immitis *in southern Italy are scant and limited to sporadic case reports. In the sole work ever conducted, out of 351 dogs parasitologically tested 63 (17.9%) were microfilariemic in the Campania region [35]. As far as identification is concerned, *A. reconditum *was the most prevalent species identified (16.5%) followed by *D. repens *(1.4%) and *D. immitis *(0.5%) [35].

In Italy, CanL caused by *L. infantum *is transmitted by different *Phlebotomus *species (Table 2[31]). For long time, stable endemic foci of CanL by *L. infantum *have been reported from central and southern areas [11,36-38] with high percentages (up to 53.1%) of serologically positive animals in southern regions [36]. Until the 1990s, CanL has been regarded as a sporadic disease in northern regions, mainly linked to animals with a history of travel to central and southern Italian regions [26].

**Table 2:** Proven or suspected Plebotominae vectors of *Leishmania infantum *in Italy (North -N; South -S; Sicily -Si; Sardinia -Sa) [23,24,31].

Species	Distribution
*Phlebotomus ariasi*	N
*Phlebotomus neglectus*	N, S, Si, Sa
*Phlebotomus perniciosus*	N, S, Si, Sa
*Phlebotomus perfiliewi*	N, S, Si

## Changing distribution patterns: one Italy for two diseases

In 1986 a review of the literature along with a questionnaire sent to public and private laboratories and clinics [39], defined as infected by *D. immitis*, 50% of the northern provinces and only 15% of the provinces of central and southern Italy. In the late 1980s and 1990s the HW infection showed a relevant prevalence increase in endemic areas compared with the 1970s [16]. Later on *D. immitis *infection was recorded outside the main endemic area of the Po Valley, in provinces of north-eastern Italy previously regarded as non-endemic [40]. Similarly, in Piedmont, an extensive survey carried out in the 1990s [34] reported a spread of both *D. immitis *and *D. repens *westward and south-westward of the traditional endemic area. In particular, *D. immitis *infection successfully established in hilly and pre-alpine areas as well in urban areas [34].

More recently, *D. immitis *has become endemic in central regions such as Tuscany, where the prevalence increased more than 7-fold in 10 years [16], and Umbria which was considered a non-endemic area until 1999 [41,42]. Nowadays, HW infection is endemic in central regions (Toscana, Umbria) with prevalences ranging from 1 to 21% [41-43]. In the latter case, similarly to that recorded in Piedmont region [34], it was observed that where an increase of prevalence and dispersion of *D. immitis *and *D. repens *was recorded, the first species spread much extensively and rapidly than *D. repens *in the same time-range. In the Lazio region, where only *D. repens *infection was recorded [43], *D. immitis *was recently found for the first time in culicid vectors [32]. Interestingly, both species have been also detected for the first time in autochthonous dogs living in another previously *Dirofilaria*-free region of central Italy, i.e. Abruzzo, close to Umbria and Lazio regions [44].

In Sardinia, a non-endemic area until 1960s, canine dirofilarioses have shown an increasing pattern of prevalence with peaks up to 17% [45] and cases have also been also reported in Sicily. [46].

While the distribution and prevalence of dirofilariosis has been widely studied in dogs from central and northern Italy, epidemiological data on the occurrence of dirofilariosis by *D. immitis *in southern Italy are scant [35] and limited to sporadic case reports. In a recent survey [47], from January 2005 to January 2008 a total of 1447 autochthonous dogs were sampled from 4 different areas of southern Italy (Apulia and Calabria regions) and grouped as follows: 404 dogs from the Bari municipal kennel for stray dogs (located in a urban area -site A), 421 dogs from the Ginosa municipal kennel for stray dogs (located in a rural area -site B), 389 owned dogs living in Bari, Lecce and Taranto municipalities, (side C) (Apulia region) and 233 dogs from the Cassano Jonico municipal kennel for stray dogs, Calabria region (site D). Animals from site C presented clinical signs related to canine HW infection while all other animals did not. All serum samples were examined by a commercial Canine Heartworm Test Kit (Idexx Laboratories^®^) to detect *D. immitis *antigens, while blood from animals of sites C and D underwent parasitological examination by modified Knott method to detect microfilariae. After serological examination, 2 animals (one for each of sites A and B) and 8 from sites C and D were diagnosed positive for the *D. immitis*. After parasitological examination, 4 animals were positive for microfilaria in both sites C and D. Hence, percentages of positivity referring to both parasitological and serological tests were: 0.24% from site A, 0.23% from site B, 2.57% from site C and 3.43% from site D. Out of the total number of 20 positive dogs (1.61% of the whole study population), 18 dogs were positive for *D. immitis *and 2 for *D. repens*.

The distribution of CanL in Italy has been revised from 1910 to 1983 [11]. Since the 1990s there has been an increase in the number of cases of CanL reported in Italy. Furthermore, new foci of CanL have been detected in northern regions, previously regarded as non-endemic [26]. The spreading of CanL northward in Italy has been assessed on the basis of recent analysis of human and dog cases of leishmaniosis recorded as well on the retrospective literature analysis of CanL. Seven leishmaniosis autochthonous foci were retrospectively identified from 1990 to 2002, whereas prospective investigations in the following years in dogs, identified other 16 possible foci all over northern regions, with low cumulative prevalence (2.1%) either from serological tests or as clinical cases [26]. Data were also confirmed by phlebotomine sandfly records with four vector species identified. In particular, *Phlebotumus perniciosus *and *Phlebotumus neglectus *were the most represented species whose population have increased in density in northern Italy when compared with historical data [26]. In north-eastern Italy, new imported cases of CanL are regularly notified outside the original area of first infection, and established foci have been recently detected [48].

## Final considerations and research needs

**Figure 3:**
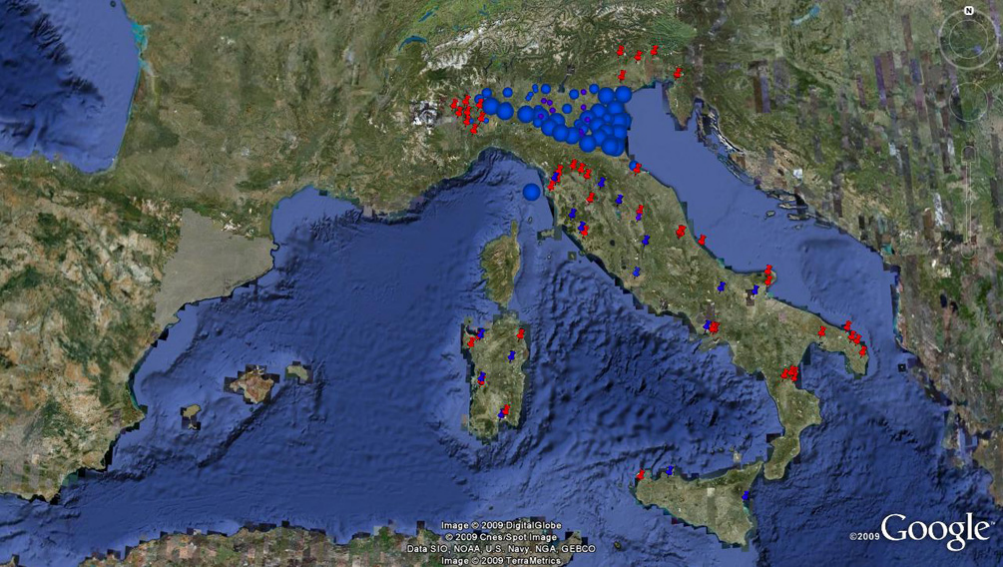
**Current distribution of *Dirofilaria immitis *in Italy**. Foci of canine dirofilariosis by *Dirofilaria immitis *until the 1990s in the endemic area of the Po Valley (blue dots) and outside the endemic area (blue pushpin). New foci (red pushpin) reported in non-endemic areas after the 1990s until 2009.

**Figure 4:**
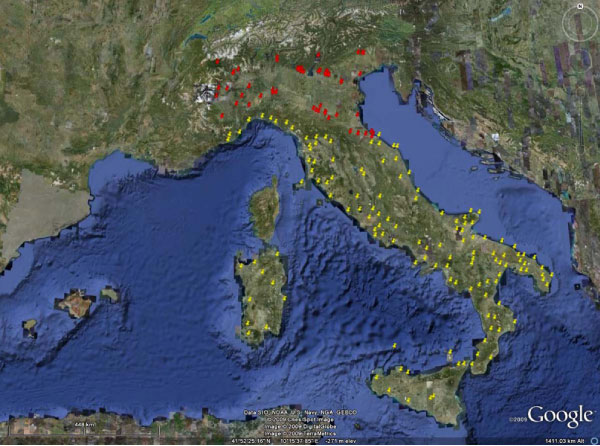
**Current distribution of *Leishmania infantum *in Italy before and after 1989**. Foci of canine leishmaniosis until the 1990s (yellow pushpin) in endemic regions of central and southern Italy (no autochthonous cases in northern regions were reported). New foci (red pushpin) in non-endemic areas after the 1990s until 2009 based on the report of autochthonous infected dogs and concomitant presence of competent sandflies.

Comparison of historical (up to 1989) and current available data (1999–2009) confirms that HW and CanL, although with different prevalence rates, have been changing their distribution patterns in Italy (Figures 3 and 4) as a result of many biological and ecological factors (e.g. vector distribution, dog movements, improved diagnostics, higher awareness of researchers and practitioners).

Thus, despite the complexity in analysing retrospective data (e.g. difficulties in retrieving local reports published on national journals, differences in diagnostic tools and methodologies employed in different surveys), the "paradigm" about the dual distribution of HW and CanL in northern and southern Italy cannot be considered valid anymore. In fact, in addition to the likely tendency toward the spreading of HW in northern countries, there has been recorded an increasing number of reports in southern Italy and islands. As a consequence, canine dirofilariosis cannot anymore be considered as confined to northeastern [17] and central regions of the peninsula [42] because of its presence in southern Italy (even if with low prevalence). Whether there will be an expansion of HW infection over future years it is difficult to predict and infection foci need to be constantly monitored.

Without any doubt, the increase in the prevalence in previous endemic areas and the colonization of contiguous environments has been due to an increased density of the vectors and to a change in the composition of mosquito population (i.e. a predominance of mosquito species more efficient in *Dirofilaria *spp. transmission). A pivotal role in this process has probably been played by spreading of *Ae. albopictus *through Italy [49]. Indeed, since its introduction in 1990 *Ae. albopictus *adapted well to the relatively low winter temperatures of Italy by rapidly increasing its populations through the country, developing insects generations over the whole year in central and southern regions and overwintering as eggs in colder northern regions [49]. The spread of *Ae. albopictus *populations, coupled with its proven role as a suitable biological vector of both *D. repens *and *D. immitis *under experimental and field conditions [32,50-52], may further account for the current wide distribution of dirofilarioses.

New epidemiological investigations should also be addressed on the occurrence of the infection in canine populations in southern Italy. In addition, entomological surveys should be carried out to estimate the occurrence of the vectors of *D. immitis *and to monitor the expansion of the small foci of dirofilariosis in the Apulia and Calabria regions that have been here presented. The above information, along with continuing veterinary education, will possibly avoid the spreading of the disease in southern Italy.

Similarly, the reports of CanL here reviewed, when compared with historical data, confirm the likely expansion of *L. infantum *infection in northern Italy which now should be considered an endemic area. Given the widespread presence of the *L. infantum *domestic reservoir and the presence of infected dogs from endemic areas (due to enhanced and facilitated animal movements from endemic to non-endemic areas) Phlebotominae vectors had probably played a main role in spreading the infection in northern area of Italy. The increasing of population density rate and the expansion of *P. perniciosus *and *P. neglectus *was confirmed by comparing recent entomological surveys with available historical data [26]. The spreading of sandfly populations has been mainly due to shortening of larval development and extension of breeding seasons as an effect of increased temperatures [53]. The above phenomena might have ultimately contributed in favouring the establishment into previously free areas or in increasing insect density into already colonized areas of phlebotomine competent species of *L. infantum *and might represent an important issue to be considered while predicting the spreading of sandflies northward through central European continental countries.

As a further issue, new tools for monitoring and diagnosing CVBD (molecular technologies, mathematical models, remote sensing and geographical information systems) may be useful in studying current and predictive distribution of pathogens [54]. As an example molecular biology tools have been used to assess the prevalence and incidence of *Leishmania *species in a given area, thus providing new information on the genetic identity of pathogens and possibly tracking the probable origin of infection in non-endemic areas [55]. Also the use of recombinant antigens for serology (e.g. rK39 for *L. infantum *and S2–S16, rWSP for *D. immitis*) have refined and implemented our understanding of the epidemiology for many CVBDs by providing new data about their distribution [54].

As a consequence, maps on the occurrence of CVBD causing pathogens in different geographical areas [56] need to be continuously updated on the basis of national level reports to assess the risk of infection spreading. The occurrence of large numbers of asymptomatic dogs in CanL [28] and HW infected populations and the long incubation periods, in which they are able to infect sandflies and mosquitoes, should be considered as an important issues when planning control measures for both diseases.

Although in Italy the perception and the awareness of researchers and practitioners on many aspects of CVBD have increased over the last decade, many issues still need to be better investigated. These include basic knowledge on vector, pathogen and host interactions which would provide new information to manage CVBD in endemic areas reducing the risk of occurrence of new foci in non endemic zones. Under the above circumstances, monitoring the disease mainly in presence of stray untreated dogs is a necessity for planning control strategies for CVBDs. While stray dogs represent an easy feeding source for arthropods and reservoir of pathogens, the scant economic resources (which are further worsened by the global economic crisis), the current legislation that obliges the health public authorities to maintain municipal kennel for stray dogs (in which animals remain often untreated against ectoparasites), probably represent the major impairments toward the control of HW and CanL in Italy as well as in other Mediterranean countries.

## Competing interests

The authors declare that they have no competing interests.
